# Comparative bioinformatic analysis suggests that specific dauer-like signalling pathway components regulate *Toxocara canis* development and migration in the mammalian host

**DOI:** 10.1186/s13071-018-3265-y

**Published:** 2019-01-14

**Authors:** Guangxu Ma, Tao Wang, Pasi K. Korhonen, Shuai Nie, Gavin E. Reid, Andreas J. Stroehlein, Anson V. Koehler, Bill C. H. Chang, Andreas Hofmann, Neil D. Young, Robin B. Gasser

**Affiliations:** 1 Department of Veterinary Biosciences, Melbourne Veterinary School, The University of Melbourne, Parkville, Victoria 3010 Australia; 2 Bio21 Molecular Science and Biotechnology Institute, The University of Melbourne, Parkville, Victoria 3010 Australia; 3 Griffith Institute for Drug Discovery, Griffith University, Nathan, Queensland 4111 Australia

**Keywords:** *Toxocara canis*, *Ascaris suum*, Dauer signalling pathway, Dafachronic acid, Arrested development

## Abstract

**Background:**

*Toxocara canis* is quite closely related to *Ascaris suum* but its biology is more complex, involving a phase of arrested development (diapause or hypobiosis) in tissues as well as transplacental and transmammary transmission routes. In the present study, we explored and compared dauer-like signalling pathways of *T. canis* and *A. suum* to infer which components in these pathways might associate with, or regulate, this added complexity in *T. canis*.

**Methods:**

Guided by information for *Caenorhabditis elegans*, we bioinformatically inferred and compared components of dauer-like signalling pathways in *T. canis* and *A. suum* using genomic and transcriptomic data sets. In these two ascaridoids, we also explored endogenous dafachronic acids (DAs), which are known to be critical in regulating larval developmental processes in *C. elegans* and other nematodes, by liquid chromatography-mass spectrometry (LC-MS).

**Results:**

Orthologues of *C. elegans* dauer signalling genes were identified in *T. canis* (*n* = 55) and *A. suum* (*n* = 51), inferring the presence of a dauer-like signalling pathway in both species. Comparisons showed clear differences between *C. elegans* and these ascaridoids as well as between *T. canis* and *A. suum*, particularly in the transforming growth factor-β (TGF-β) and insulin-like signalling pathways. Specifically, in both *A. suum* and *T. canis*, there was a paucity of genes encoding SMAD transcription factor-related protein (*daf-3*, *daf-5*, *daf-8* and *daf-14*) and insulin/insulin-like peptide (*daf-28*, *ins-4*, *ins-6* and *ins-7*) homologues, suggesting an evolution and adaptation of the signalling pathway in these parasites. In *T. canis*, there were more orthologues coding for homologues of antagonist insulin-like peptides (*Tc-ins-1* and *Tc-ins-18*), an insulin receptor substrate (*Tc-ist-1*) and a serine/threonine kinase (*Tc-akt-1*) than in *A. suum*, suggesting potentiated functional roles for these molecules in regulating larval diapause and reactivation. A relatively conserved machinery was proposed for DA synthesis in the two ascaridoids, and endogenous Δ4- and Δ7-DAs were detected in them by LC-MS analysis. Differential transcription analysis between *T. canis* and *A. suum* suggests that *ins-17* and *ins-18* homologues are specifically involved in regulating development and migration in *T. canis* larvae in host tissues.

**Conclusion:**

The findings of this study provide a basis for functional explorations of insulin-like peptides, signalling hormones (i.e. DAs) and related nuclear receptors, proposed to link to development and/or parasite-host interactions in *T. canis*. Elucidating the functional roles of these molecules might contribute to the discovery of novel anthelmintic targets in ascaridoids.

**Electronic supplementary material:**

The online version of this article (10.1186/s13071-018-3265-y) contains supplementary material, which is available to authorized users.

## Background

*Toxocara canis* is an important pathogen of both animal and human health importance worldwide [1]. This parasite, which is related to other ascaridoid nematodes such as *Ascaris* spp., can be directly transmitted to the human host *via* a faecal-oral route, and can cause toxocariasis and complications such as neurological and allergic diseases [1].

The biology of *T. canis* is complex and involves canids (e.g. dogs, wolves and foxes) as definitive hosts, paratenic hosts such as rodents, and accidental hosts including humans [2, 3]. The eggs of this parasite are expelled in the faeces from canids, embryonate and become infective in the environment. Following the ingestion of infective eggs by the canid host, infective, third-stage larvae (L3s) emerge from the eggs, penetrate the intestinal wall and migrate to the liver and lungs (hepato-pulmonary migration). In young dogs (< 12 weeks), larvae migrate to the airways and then get swallowed and make their way to the small intestine, where fourth-stage larvae (L4s) develop to adult worms (female and male), mate and reproduce. In dogs of ≥ 12 weeks, oral infection can occur, but larvae tend to encyst in various tissues (including muscles, brain and nerves), where they undergo hypobiosis (i.e. arrested development or diapause). In female dogs, encysted larvae become activated (in the last trimester of pregnancy), most of which undergo transplacental transmission to the foetuses in utero (~99%), and a minority of which (~1%) undergo transmammary (transcolostoral) transmission to newborn pups [4]. Adult worms eventually establish in the small intestine of pups, and reproductively-active female worms are a major source of egg contamination. *Toxocara canis* can also be transmitted to accidental (paratenic) hosts (e.g. rats, mice and rabbits) *via* accidental ingestion of infective eggs; in such hosts, larvae hatch from the eggs, penetrate the intestinal wall and then migrate to various organs and tissues, where they cause disease and/or encyst. If a canid eats infected tissues (containing larvae) from such a paratenic host, adult worms can develop in the small intestine [2, 3].

Humans are accidental hosts. They can become infected by ingesting infective eggs from contaminated soil, food or water, or larvae in tissues from infected paratenic hosts [1]. Following ingestion, infective larvae are released and invade the intestinal wall, and are then carried *via* the blood circulation to various tissues (including liver, lungs, muscles, central nervous system). Although these larvae undergo arrested development in these tissues, during prior migration they frequently cause pathogenic effects due to associated mechanical damage, inflammatory responses and granuloma formation, leading to the disease toxocariasis, of which there are four main clinical forms (visceral larva migrans, ocular larva migrans, neurotoxocariasis and covert toxocariasis) [1, 5]. Such larvae in tissues have also been implicated in neurodegenerative disorders (e.g. epilepsy, idiopathic Parkinson’s disease and dementia) and in allergic diseases (e.g. asthma and pruritus) [6–10].

Interestingly, *T. canis* has a marked tropism for the central nervous system (cerebrum) [11]. Larvae that migrate through the brain downregulate lipid/cholesterol biosynthesis [12]. There is evidence that prolactin (pituitary hormone) plays a role in activating arrested larvae of *T. canis* [13], and some studies have implicated this hormone in regulating larval growth and motility in *T. canis*, infection intensity and host immune responses [14, 15]. The information from these studies indicates that lipid or hormone signalling plays critical roles in the migration, diapause and host interplay of *T. canis* larvae. Roles of similar signalling pathways in regulating developmental processes have been described for the free-living nematode *Caenorhabditis elegans* [16–18]. Specifically, steroid hormone signalling, particularly the dafachronic acid-DAF-12 module, has been recognised as a ‘check-point’ for diapause (dauer) in this worm [19–22], and has been proposed to relate to an endocrine mechanism which appears to be conserved between free-living and parasitic nematodes [23–26]. However, little is known about the signalling pathways in parasitic nematodes and the differences in such pathways between *Toxocara* and other ascaridoids such as *A. suum* [27], which, unlike *T. canis*, does not undergo transplacental or transmammary transmission [28, 29]. Profound knowledge of dauer and associated signalling pathways in *C. elegans* enables comparative studies in parasitic nematodes utilising transcriptomic and genomic data sets and tools [30, 31], in the absence of functional genomic data. Here, guided by information and data for *C. elegans*, we inferred dauer-like signalling pathways of *T. canis* and *A. suum*, and identified unique components that we hypothesise regulate the development and/or other molecular processes in *T. canis* in the mammalian host.

## Methods

### Draft genomes and transcriptomes

Genome assemblies and annotations for *C. elegans* (BioProject PRJNA13758), *T. canis* (BioProjects PRJEB533 and PRJNA248777) and *A. suum* (BioProjects PRJNA80881 and PRJNA62057) were obtained from WormBase [32] and ParaSite at WormBase [33]. Transcriptomic data sets of *T. canis* and *A. suum* (BioProjects PRJNA248777 and PRJNA80881) were taken from NCBI Sequence Read Archive (SRA; https://www.ncbi.nlm.nih.gov/sra) [34–37]. New versions of the transcriptomes of *T. canis* and *A. suum* were assembled *de novo* using the program Trinity v.2.4.0 [38, 39].

### Identification of dauer signalling gene homologues

Genes (*n* = 107) representing the canonical dauer signalling pathway [i.e. cyclic guanosine monophosphate (cGMP), transforming growth factor-β (TGF-β), insulin/insulin-like growth factor (IGF), and steroid hormone signalling pathways] in *C. elegans* were available from published information [19, 27, 40]. Protein sequences (*n* = 182) and functional information were obtained from WormBase (WS261) [41]. Homologues were predicted by exhaustive homology searching of *C. elegans* protein sequences (using BLAT v.35 and tblastn v.2.5.1) against the genome and transcriptome assemblies of *T. canis* and *A. suum*. In addition, Pfam, PANTHER and SUPERFAMILY conserved domain architectures (using InterProScan v.5.15.54) [42, 43] of the dauer signalling gene products were used to predict homologues from the original gene predictions and *de novo*-assembled transcripts of these two ascaridoid species, using an established approach [44]. Predicted transcripts of potential homologues were matched (using blastx v.2.5.1, *e*-value: ≤ 10^-5^) to proteins of *C. elegans* (BioProject PRJNA13758.WS261) to verify their identity.

### Curation of genes and classification of orthologues

Homologous sequences were manually curated using a recently described workflow [44]. In brief, identified gene and transcript sequences were mapped to the genome assemblies of each *T. canis* and *A. suum* using the program BLAT v.35 [45]. Transcripts that mapped to the same coding region were re-assembled using the program CAP3 for possible extensions [46]. Full-length transcripts were used to refine the corresponding gene models using the program Exonerate v.2.2.0 [47]. Gene products were predicted from the curated coding DNA sequences (CDSs) using ORFfinder [48]. Gene orthologues were classified according to groups inferred by OrthoMCL (*e*-value: ≤ 10^-5^; sequence similarity: ≥ 30%) [49].

### Comparative analyses

Sequence similarities of the classified orthologues were compared by pairwise alignment using EMBOSS Needle (the Needleman-Wunsch algorithm) [50]. Domain architectures of inferred proteins were assigned using InterProScan v.5.15.54. Specifically, homologues of genes encoding insulin-like peptides were identified based on their sequence domain signatures. The inferred protein sequences were compared with the insulin/insulin-like peptides of *C. elegans* using blastp (*e*-value ≤ 10^-5^) to infer their identity. For inferred insulin-like peptides, conserved patterns and motifs were searched using the programs Pratt v.2.1 [51] and MEME v.5.0.2 [52]. The relationships of insulin-like peptides were verified manually using OrthoMCL (*e*-value: ≤ 10^-5^; sequence similarity: ≥ 50%).

### Transcriptional analysis

Available RNA-seq reads from egg, and first- (L1), second- (L2), third- (L3, recovered from eggs, liver and lungs) and fourth-stage larvae and/or adults of *T. canis* and of *A. suum* [34–36] were used for transcriptional analyses and comparisons. In brief, paired-end reads were mapped to individual curated CDSs using Bowtie2 v.2.1.0 within the software package RSEM v.1.2.11 [53, 54]. Levels of messenger RNA (mRNA) transcription were recorded in transcripts per million (TPM). For individual developmental stages, transcription profiles for individual orthologues were displayed in a heat-map using the program heatmap.2 (in R v.3.5.1).

### Liquid chromatography-mass spectrometry (LC-MS)

For each *T. canis* and *A. suum*, lipids were extracted from four individual male and four individual female adults using an established lipid extraction method [55, 56]. In brief, individual samples (1 mg dry weight; 4 replicates) were suspended in ice cold 40% methanol and homogenised using zirconium oxide beads (ZROB05, Next Advance, USA). A chloroform:methanol (2:1) mix was used to separate the aqueous and organic phases by centrifugation at 10,000× *g* for 10 min at room temperature (24 °C). The organic phase was retained, dried and resuspended in methanol for subsequent mass spectrometric analysis using an Orbitrap Fusion Lumos mass spectrometer coupled to an Ultimate 3000 UHPLC (Thermo Fisher Scientific, San Jose, CA, USA). Commercially available dafachronic acids (25S)-Δ7-DA and (25S)-Δ4-DA (exact mass: 413.3061) (cat no. 23017-97-2; Cayman Chemical Company) were used as reference standards for the identification of endogenous DAs.

## Results

### Dauer signalling orthologues

**Fig. 1:**
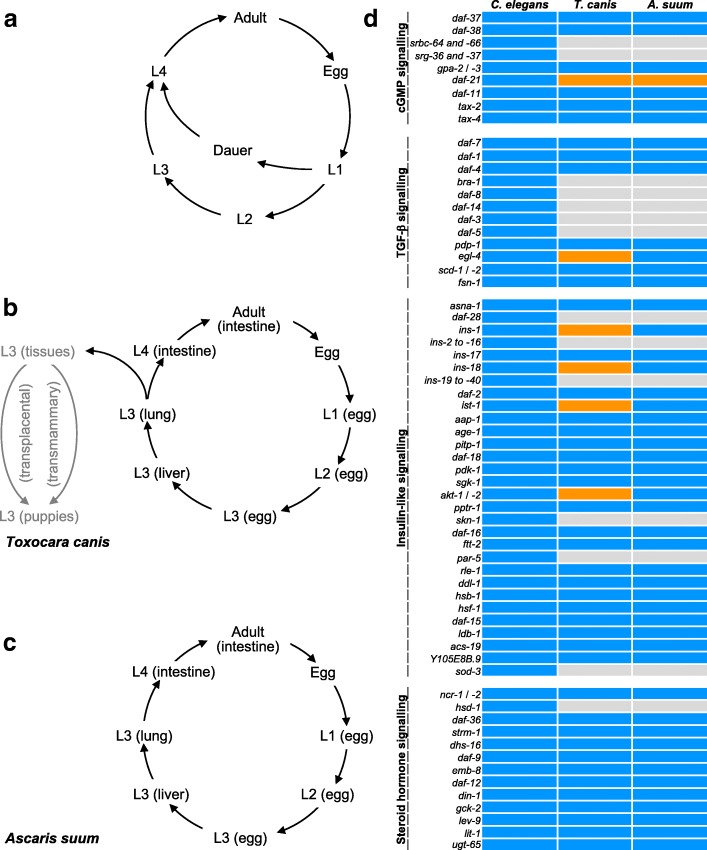
Life-cycle and dauer signalling orthologues of *Caenorhabditis elegans*, *Toxocara canis* and *Ascaris suum*. Schematic representation of the life-cycles of *C. elegans* (**a**), *T. canis* (**b**) and *A. suum* (**c**). Specifically, the third-stage larvae (L3s) of *T. canis* can arrest in the tissues for years, and can be reactivated in the female dog (bitch) in the last trimester of pregnancy and then migrate to uterus or mammary glands (*post partum*), leading to transplacental or transmammary transmission to offspring (grey arrows). **d** Dauer signalling gene orthologues in *T. canis* and *A. suum* are indicated (in blue) and compared to *C. elegans* genes. Increased numbers of orthologues are identified in *T. canis* and/or *A. suum* (orange). Orthologues not inferred for *T. canis* and/or *A. suum* are indicated in grey

Based on the information available for *C. elegans*, we identified 55 and 51 orthologues encoding signalling molecules in *T. canis* and *A. suum*, respectively (Additional file 1: Tables S1-S4). These numbers are markedly lower than for *C. elegans* (*n* = 107) and relate mainly to less TGF-β and insulin/insulin-like signalling components in the ascaridoids. Specifically, orthologues inferred to represent SMAD transcription factors (*daf-3, daf-8* and *daf-14*), SKI family transcriptional co-repressor (*daf-5*), insulin (*daf-28*), insulin-like peptides (e.g. *ins-4*, *ins-6* and *ins-7*), serine/threonine-protein kinase (*akt-2*), bZip transcription factor (*skn-1*), 14-3-3 protein (*par-5*) and iron/manganese superoxide dismutase (*sod-3*) were not detected in either *T. canis* or *A. suum*, whereas two orthologues encoding heat-shock protein 90 (*daf-21*) were identified in both species (Fig. 1d). A comparison indicated more orthologues coding for a cGMP-dependent protein kinase, insulin-like peptides, an insulin receptor substrate and a serine/threonine-protein kinase (*egl-4*, *ins-1*, *ins-18*, *ist-1* and *akt-1*) in *T. canis* compared with *A. suum* (see Fig. 1d). More transcript isoforms were predicted for *T. canis* orthologues, such as *Tc-akt-1* (*n* = 5) and *Tc*-*daf-12* (*n* = 18), than for *A. suum* (see Additional file 1: Tables S3 and S4).

### Insulin-like peptide-coding genes and their relationships

**Fig. 2:**
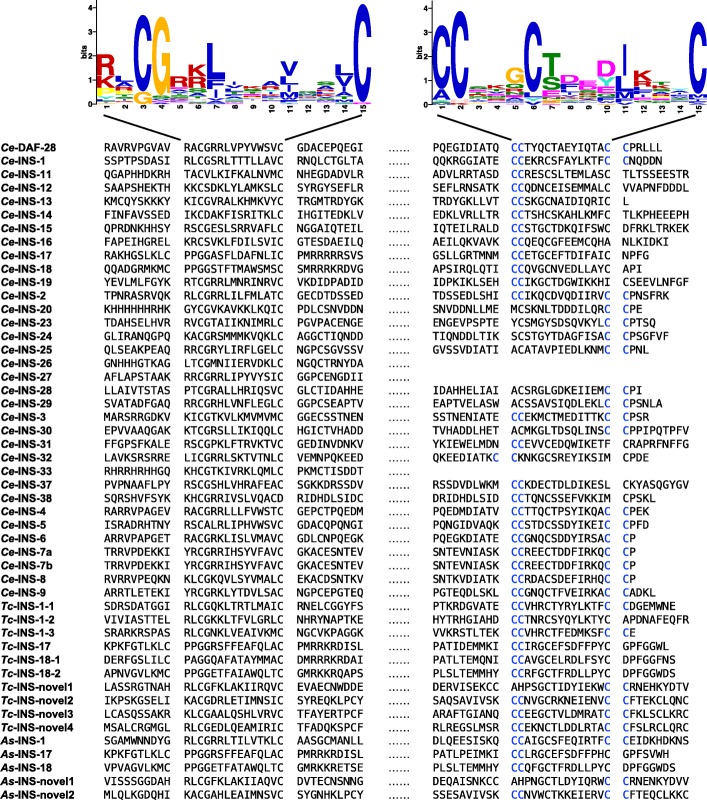
Motifs and conserved pattern of insulin-like peptides inferred for *Caenorhabditis elegans*, *Toxocara canis* and *Ascaris suum*. Motifs RLCGRKLIKAVQSLC and CCSKGCTDEDJKKYC in the inferred protein sequences are aligned and indicated in sequence logos. The conserved pattern C-C in these sequences is indicated in blue

We identified 10 and 5 sequences encoding signatures characteristic of the insulin-like superfamily in *T. canis* and *A. suum*, respectively (Additional file 1: Table S5). Although marked sequence diversity (23-77%) was seen among the inferred insulin-like peptides of *C. elegans*, *T. canis* and *A. suum* (Additional file 1: Table S5), two characteristic motifs (RLCGRKLIKAVQSLC and CCSKGCTDEDJKKYC; *P*-value: ≤ 10^-5^) and one conserved sequence pattern (C-C; fitness = 8.34) were discovered (Fig. 2). Specifically, the proteins inferred for *T. canis* and *A. suum* had significant sequence similarity (blastp *e*-value cut-off: ≤ 10^-5^) to *Ce*-INS-1, *Ce*-INS-12, *Ce*-INS-17, *Ce*-INS-18 or *Ce*-INS-32 (Additional file 1: Table S5). Relationships among the insulin-like peptides for these species were supported by their orthologous groups (*n* = 12; *e*-value: ≤ 10^-5^; sequence similarity: ≥ 50%) (Additional file 1: Table S5). Apart from the orthologues inferred (i.e. INS-1, INS-17 and INS-18), one more homologue of *Ce-ins-1* was inferred for *T. canis*, and novel insulin-like peptides were predicted for *T. canis* (*n* = 4) and *A. suum* (*n* = 2) (Fig. 2; Additional file 1: Table S5).

### Dafachronic acid biosynthesis machinery

**Fig. 3:**
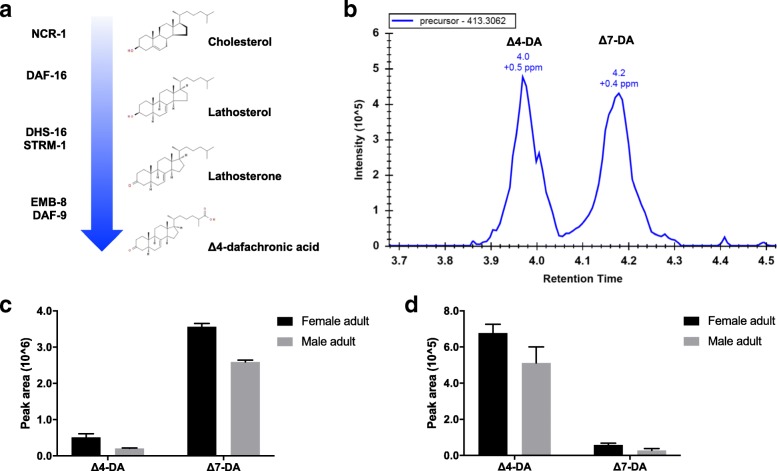
Dafachronic acids (DAs) in *Toxocara canis* and *Ascaris suum*. **a** Proposed biosynthetic pathway of Δ7-DA in *T. canis* and *A. suum*, which involves NCR-1, DAF-36, DHS-16, STRM-1, EMB-8 and DAF-9. **b** Using Δ4- and Δ7-DAs (exact mass = 413.3061) as standards, endogenous DAs, with mass errors estimated at 0.5 and 0.4 parts per million (ppm), are present in the adults (female and male) of *T. canis* and *A. suum*, respectively. **c** and **d** Relative abundances of Δ4- and Δ7-DAs in the adult (female and male) stages of *T. canis* and *A. suum*, respectively

We inferred 6 orthologues to be involved in the biosynthesis of DAs. Specifically, the identification of orthologues *ncr-1*, *daf-36*, *dhs-16*, *strm-1*, *emb-8* and *daf-9* indicated a relatively conserved biosynthetic pathway for DA (i.e. Δ7) among *C. elegans*, *T. canis* and *A. suum* (Fig. 3a), although the orthologue of *Ce-hsd-1* was not identified in either *T. canis* or *A. suum*.

Endogenous DAs (Δ4 and Δ7) were detected by LC-MS in the female and male adults of *T. canis* and *A. suum*, with mass errors estimated at 0.5 and 0.4 parts per million (ppm), respectively (Fig. 3b). Specifically, Δ7-DA was dominant in *T. canis*, whereas Δ4-DA was in *A. suum* (Fig. 3). For both species, the relative abundance of DAs in female adults was higher than in male adults (*P* > 0.05) (Fig. 3c).

### Developmental transcription of dauer signalling components

**Fig. 4:**
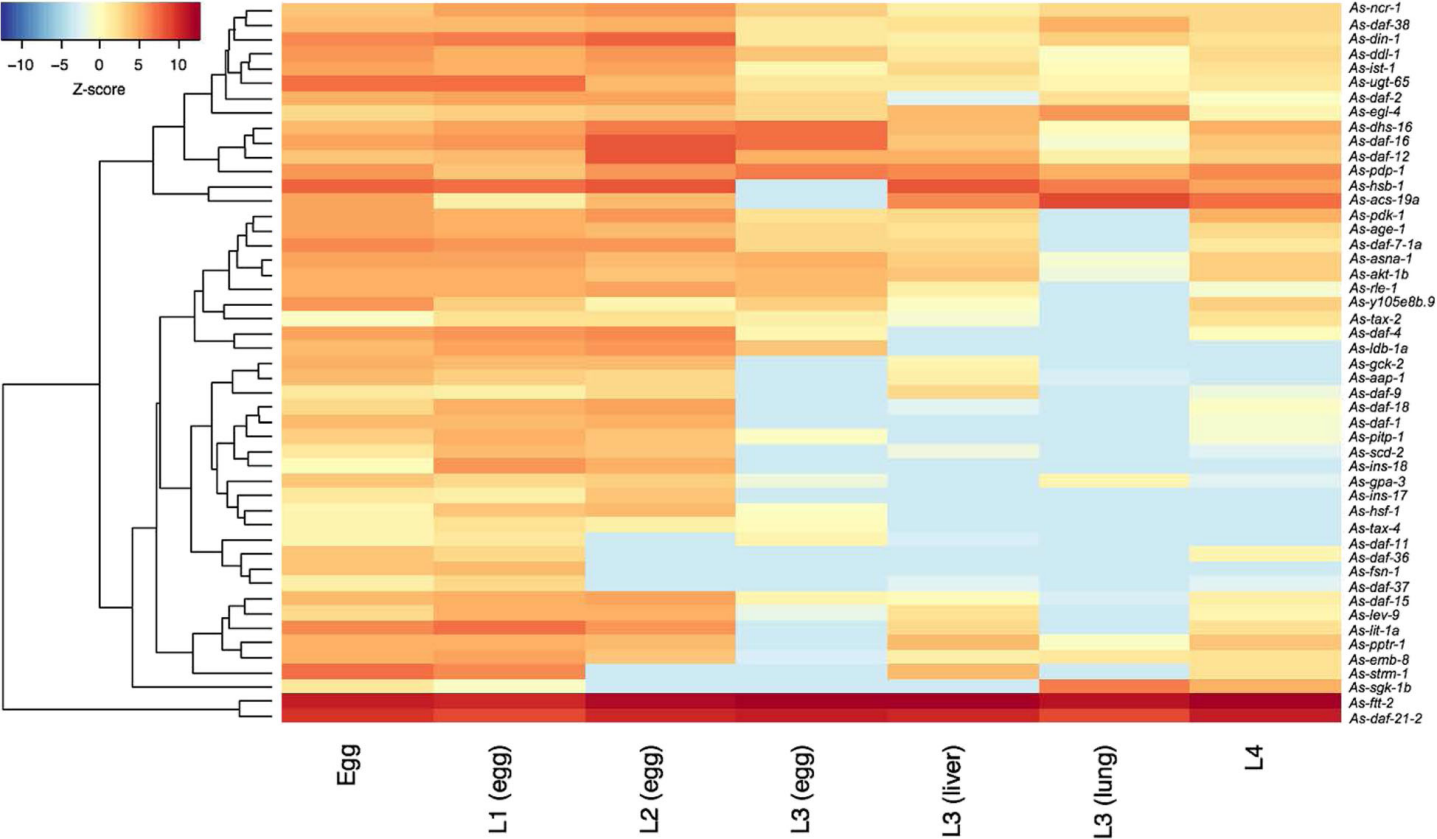
Transcriptional profiles of dauer signalling genes in different developmental stages of *Ascaris suum*. Transcriptional levels (transcripts per million, TPM) of dauer signalling orthologues in egg, the first- and second-stage larvae recovered from eggs; third-stage larvae from eggs, host liver or lung, and the fourth-stage larvae of *A. suum* are indicated in the heat map. Colour scales and Z-scores indicate scaled TPMs

We compared levels of mRNA transcription of individual dauer signalling orthologues in L3 and adult (female and male) stages of *T. canis* (Additional file 1: Table S6). For some orthologues, including *Tc-gpa-3*, *Tc-daf-11* and *Tc-tax-4* (cGMP signalling), *Tc-asna-1* and *Tc-ins-18* (insulin-like signalling), and *Tc-daf-9* (steroid hormone signalling), transcription was higher in the L3 than the adult stage (Additional file 1: Table S6). We also compared mRNA transcription levels of individual dauer signalling orthologues in 7 distinct developmental stages of *A. suum* (Additional file 1: Table S6). Although similar transcriptional profiles were observed in egg and L1 stages, marked variation was seen among L2, L3 (recovered from eggs, liver and lungs) and L4 stages of *A. suum*, particularly for the genes *As-gpa-3*, *As-daf-11* and *As-tax-4* (cGMP signalling), *As-asna-1* and *As-daf-2* (insulin-like signalling), and *As-daf-9* (steroid hormone signalling) (Fig. 4). A comparative analysis showed high levels of transcription for the orthologues *daf-21* and *ftt-2* in the stages of *T. canis* and *A. suum* studied, and low levels for *ins-17* and *ins-18* in the latter species (Additional file 1: Table S6).

## Discussion

Based on current genomic and transcriptomic datasets, we identified orthologues of the dauer signalling genes in *T. canis* and compared them with those of *A. suum* using a bioinformatic strategy for gene curation and classification. We compared these components, proposed the biosynthetic pathways of DA and investigated developmental transcription of identified orthologues.

The identification of key dauer-associated signalling gene orthologues implies dauer-like signalling pathways in *T. canis* and *A. suum*. Although dauer signalling gene homologues had been identified previously in a range of parasitic nematodes, including *A. suum* and *T. canis* [27], orthologues were defined here using an effective approach for curation and classification [44]. Specifically, the sequence re-assembly strategy used increased the likelihood of identification. For instance, we identified orthologues of *Ce-ins-1* and *Ce-dhs-16* in both *T. canis* and *A. suum*, which were not reported previously [27]. In addition, the present sequence-based classification strategy improved the accuracy of annotation. Although *Ce-bra-1* homologues had been identified [27], the orthologue of this gene was not identified herein in *T. canis* or *A. suum*. Specifically, orthologues of *Ce-ftt-2* and *Ce-par-5* (encoding 14-3-3 proteins) were classified based on reciprocal BLAST searches and OrthoMCL grouping. Thus, the accurate identification and classification of orthologues provided a basis for reliable comparative studies.

The present findings indicated a divergent evolution and adaptation of the dauer-like signalling pathway among the three nematodes studied. Compared with *C. elegans*, the reduced number of genes coding for SMAD-related transcription factors and insulin/insulin-like peptides in *T. canis* and *A. suum* suggest divergences in both TGF-β and insulin-like signalling between the free-living and the two parasitic nematodes. Specifically, orthologues of *Ce-daf-3*, *Ce-daf-5*, *Ce-daf-8* and *Ce-daf-14* were not detected in *T. canis* or *A. suum*, suggesting a uniqueness in TGF-β signalling in these two species (clade III), as suggested previously by other workers for other parasitic nematodes such as *Trichinella spiralis* and *Trichuris suis* (clade I) [27, 57]. In addition, apart from *ins-1*, *ins-17* and *ins-18*, no orthologue of any of the other insulin/insulin-like peptide-coding genes (*n* = 37) of *C. elegans* (clade V) was found, implying a contraction of the gene family representing these signalling molecules in the two ascaridoids (clade III) studied here. Reduced numbers of genes encoding insulin-like peptides have been reported also in other parasitic nematodes, including *Strongyloides stercoralis* (clade IV) [40].

Distinctiveness in signalling between *T. canis* and *A. suum* might relate to differences in biology and/or developmental regulation. Although the dauer-like signalling pathways of *T. canis* and *A. suum* were similar, the increased number of orthologues in the former species indicates a distinction in TGF-β and insulin-like signalling (Fig. 1d) which might associate with entry into and exit from arrested larval development in *T. canis*. First, the three *Tc-ins-1* and two *Tc-ins-18* paralogues encode antagonist insulin-like peptides, which are recognised to function in promoting dauer formation in *C. elegans* [58, 59]. The novel insulin-like peptides (*n* = 4; *Tc-ins-1n* to *Tc-ins-4n; n = novel*) predicted for *T. canis* might also be involved in regulating larval development, but this proposal needs to be assessed. Secondly, the two *Tc-ist-1* paralogues likely encode insulin receptor substrates, which function together with phosphoinositide 3-kinase (PI3K) adaptor/regulatory subunit to potentiate dauer-associated *daf-2*/insulin-like signalling, as known for *C. elegans* [60]. Thirdly, the two *Tc-akt-1* orthologues encode serine/threonine kinases Akt/protein kinase B, which play a role in antagonising the fork head transcription factor DAF-16 when activated by phospholipid products from PI3K [61]. Therefore, compared with *A. suum*, it appears that *T. canis* produces more antagonist molecules (INS-1 and INS-18) which may trigger a stronger inhibitive effect on the fork head transcription factor DAF-16 (*via* phosphorylation of a serine/threonine kinase) to enable arrested development, particularly when migrating through and/or encysting in host tissues. In addition, the relatively high numbers of transcript isoforms for *Tc-akt-1* (*n* = 5) and *Tc-daf-12* (*n* = 18) might indicate rapid adaptive expression and/or plastic functional roles, similar to those of *daf-16* reported for *C. elegans* [62]. However, although two *Tc-egl-4* paralogues were identified in *T. canis*, their functions are unclear, because orthologues coding for SMAD-related transcription factors [63] were not identified in this nematode. Clearly, detailed functional explorations of these molecules need to be undertaken to explain their roles in the development, migration and/or hypobiosis of *T. canis* larvae. Significant progress has been made through the development of a functional genomics platform for *A. suum* [64], which suggests that a similar system might be established for *T. canis*, in order to explore the functional roles of signalling molecules in this species.

To investigate dauer-like signalling pathway components in *T. canis* and *A. suum*, we also analysed transcription profiles across developmental stages, employing RNA-sequence data for pooled worms. The consistently high messenger RNA levels of *daf-21* and *ftt-2* suggest key roles for these genes and their products in larval development, supported, to some extent, by information for *C. elegans* [19]. By contrast, variable transcription levels for the genes *gpa-3*, *daf-11* and *tax-4* (cGMP signalling), *asna-1* (insulin-like signalling) and *daf-9* (steroid hormone signalling) among developmental stages, particularly L3 stages in eggs, liver and lung, might suggest plastic, but crucial roles in regulating developmental processes and/or host-parasite interactions. In addition, differential transcriptional levels of the genes *ins-17* and *ins-18* in the L3 stage between *T. canis* and *A. suum* suggest a distinctive signalling mechanism in the host animal, since *Ce*-*ins-17* and *Ce*-*ins-18* promote dauer formation in *C. elegans* [59, 65]. This difference might suggest unique roles for selected insulin-like peptides in regulating arrested development in host tissues or parasite-host interactions [57, 66]. This hypothesis warrants testing through large-scale, integrative ‘omic investigations.

Comparisons with *C. elegans* implied relative conservation in the biosynthetic machinery for DA between *T. canis* and *A. suum*. The orthologues of *Ce-ncr-1*, *Ce-daf-36*, *Ce*-*dhs-16*, *Ce*-*emb-8* and *Ce*-*daf-9* likely function in the trafficking, catalysation and modification of cholesterol destined for the biosynthesis of DAs, known to be critical for regulating larval development in *C. elegans* and *S. stercoralis* [16, 67–69]. The biosynthetic machinery proposed for ascaridoids is supported by the identification of endogenous Δ4- and Δ7-DAs in *T. canis* and *A. suum*. Interestingly, although Δ4- and Δ7-DAs are isomers, differential abundance in the adult stages of *T. canis* and *A. suum* might suggest functional distinctions in specific biological processes between these species. Based on the information for *C. elegans* and some parasitic nematodes [40, 70], the endogenous biosynthesis of Δ7-DA might be regulated by the *strm-1* gene in *A. suum*. Although similar components of the DA synthesis were predicted for both *T. canis* and *A. suum*, the functionality of this machinery in these species needs to be shown. By contrast, an orthologue of *Ce-hsd-1*, which likely functions in the biosynthesis of other DAs, was not detected in either *T. canis* or *A. suum* [71], suggesting that both of these ascaridoid nematodes have a simplified machinery to synthesise these signalling hormones. Although it seems that there is a relatively conserved steroid hormone signalling module in these ascaridoids, future work should focus on verifying the biosynthesis signalling module and the functional roles of DAs in these worms. Specifically, although it has been reported that prolactin plays a role in re-activating arrested larvae of *T. canis* [13], the proposed role for DAs (“bile acid-like steroids”) in larval reactivation (relating particularly to ensuing transplacental and/or transmammary transmission) and their associated signalling pathway(s) remain to be explored [21, 68, 72]. A better understanding of these areas, particularly signalling hormones and their nuclear receptors, would provide insight into regulatory processes in larval development and might enable the discovery of new anthelmintic targets [22, 73, 74].

## Conclusions

The present study reveals distinctiveness in the TGF-β and insulin-like signalling pathways among *C. elegans*, *T. canis* and *A. suum*, but indicates similarity in the steroid hormone signalling pathway between the two ascaridoid nematodes. Inferring the elements of the dauer-like signalling pathway in these ascaridoids provides a basis for future explorations of the functional roles of these elements in each species using a reliable functional genomics platform. Understanding these processes could enlighten developmental processes and host-parasite interactions for these ascaridoids and facilitate the discovery of novel intervention strategies against the diseases that these parasites cause.

## Additional file

Additional file 1: **Table S1.** Dauer signalling gene homologues of *Toxocara canis* and *Ascaris suum*. **Table S2.** Orthologous groups of dauer signalling genes in *Toxocara canis* and *Ascaris suum* inferred from *Caenorhabditis elegans*. **Table S3.** Salient information on dauer signalling genes of *Toxocara canis* predicted from *Caenorhabditis elegans* orthologues. **Table S4.** Salient information on dauer signalling genes of *Ascaris suum* predicted from *Caenorhabditis elegans* orthologues. **Table S5.** Classification of insulin-like peptide homologues in *Toxocara canis* and *Ascaris suum*. **Table S6.** Transcription of dauer-like signalling gene orthologues in key developmental stages of *Toxocara canis* and *Ascaris suum*. (XLSX 418 kb)

## Abbreviations

DAs: Dafachronic acids
LC-MS: Liquid chromatography-mass spectrometry
TGF-β: Transforming growth factor-β
cGMP: Cyclic guanosine monophosphate
IGF: Insulin/insulin-like growth factor
TPM: Transcripts per million
PI3K: Phosphoinositide 3-kinase
